# IgY Targeting Bacterial Quorum-Sensing Molecules in Implant-Associated Infections

**DOI:** 10.3390/molecules25174027

**Published:** 2020-09-03

**Authors:** Ulrike Dapunt, Birgit Prior, Christopher Oelkrug, Jan Philippe Kretzer

**Affiliations:** 1 Center for Orthopedics, Trauma Surgery and Spinal Cord Injury, Heidelberg University Hospital, Schlierbacher Landstrasse 200a, 69118 Heidelberg, Germany; 2 Department of Anesthesiology, Heidelberg University Hospital, Im Neuenheimer Feld 672, 69120 Heidelberg, Germany; Birgit.Prior@immu.uni-heidelberg.de; 3 Oelkrug Enterprises UG (haftungsbeschraenkt), Gerhart Hauptmann Str. 10, 59387 Ascheberg, Germany; christopher.oelkrug@oelkrug-enterprises.com; 4 Laboratory of Biomechanics and Implant Research, Center for Orthopedics, Trauma Surgery and Spinal Cord Injury, Heidelberg University Hospital, Schlierbacher Landstrasse 200a, 69118 Heidelberg, Germany; Philippe.Kretzer@med.uni-heidelberg.de

**Keywords:** implant-associated infections, biofilms, quorum-sensing molecules, GroEL, AtlE, PIA, IgY

## Abstract

*Background*: Implant-associated infections are still a major complication in the field of orthopedics. Bacteria can form biofilms on implant surfaces, making them more difficult to detect and treat. Since standard antibiotic therapy is often impaired in biofilm infections, particular interest is directed towards finding treatment alternatives. Biofilm-formation is a well-organized process during which bacteria communicate via quorum-sensing molecules (QSM). The aim of this study was to inhibit bacterial communication by directing avian IgY against specific QSM. *Methods*: Chicken were immunized against the following QSM: (1) AtlE, a member of the autolysin family which mediates attachment to a surface in *Staphylococcus epidermidis*; (2) GroEL, the bacterial heat shock protein; (3) PIA (polysaccharide intercellular adhesion), which is essential for cell–cell adhesion in biofilms. *Staphylococcus epidermidis* biofilms were grown and inhibition of biofilm-formation by IgYs was evaluated. Additionally, human osteoblasts were cultivated and biocompatibility of IgYs was tested. *Results*: We were able to demonstrate that all IgYs reduced biofilm-formation, also without prior immunization. Therefore, the response was probably not specific with regard to the QSM. Osteoblasts were activated by all IgYs which was demonstrated by microscopy and an increased release of IL-8. *Conclusions*: In conclusion, avian IgY inhibits biofilm-formation, though the underlying mechanism is not yet clear. However, adverse effects on local tissue cells (osteoblasts) were also observed.

## 1. Introduction

Implant-associated infections are still one of the most severe complications in the field of orthopedic surgery. Patients often require multiple revision surgeries and suffer from a prolonged course of treatment ([1], reviewed in [2,3,4]). These infections are particularly difficult to diagnose and to treat, because bacteria attach to the implant surface and embed themselves in a slimy matrix, the so-called extracellular polymeric substance. By doing so, they form a biofilm-colony [5,6]. Biofilms have been investigated extensively, initially with regard to industrial water-based processes such as water distribution or the operation of paper mills or cooling towers. Damages caused by these bacterial colonies are referred to as “biofouling” and amount to billions of dollars per year [7]. More recently, it has been acknowledged that biofilms are probably the most common cause for persistent bacterial infections in humans, in particular in association with foreign material (i.e., cardiac pacemakers, central venous catheter, orthopedic implants) [5].

Biofilm formation is a well-organized, genetically driven process that offers bacteria protection from adverse environmental conditions or lack of nutrition [8,9,10]. In the human body, however, bacteria additionally face the host’s defense mechanisms, which most likely plays a central role in driving opportunistic bacteria to acquire pathogenic potential and defense strategies. Therefore, it has been proposed that biofilm communities are the preferred form of living for bacteria and that planktonic (single cell) bacterial growth mainly occurs under artificial culture conditions; circumstances under which bacteria do not feel threatened in any way [11,12,13].

In order to facilitate biofilm formation, bacteria communicate via quorum sensing molecules (QSM), also known as autoinducers. Each bacterial cell produces QSM and at the same time measures the concentration of QSM in the vicinity. As the bacterial population grows, so does the concentration of QSM and once a certain threshold is reached, bacterial gene expression is altered and directed towards a common goal, such as forming a biofilm colony [14,15,16]. Several of these molecules have been studied in depth and three general quorum-sensing systems have been described [15]. First, the LuxI/R quorum sensing system has been typically attributed to Gram-negative bacteria. The autoinducer AHL (acylated homoserine lactone) is produced by LuxI enzymes and binds to LuxR proteins which leads to transcription of target genes [17]. AHL-12, in particular, does not merely serve as an autoinducer, but has been termed an interkingdom-signaling molecule, because it also interacts with mammalian cells. We were able to demonstrate in our previous work that AHL-12 activates defense-relevant functions of phagocytic cells which favors the hypothesis that immune cells eavesdrop on bacterial communication in order to recognize and attack biofilm formation early on [18]. Gram-positive bacteria use a two-component system. Modified oligopeptides serve as autoinducers which in high concentrations bind to their receptor and activate a kinase. This leads to phosphorylation of transcription factors which control quorum-sensing target genes. The third group of quorum-sensing systems is a hybrid between Gram-negative and Gram-positive systems. One of the autoinducers is an AHL (as in Gram-negative systems), but as in Gram-positive systems, signal transduction is facilitated by a two-component phosphorylation process [15].

Staphylococcus species were shown to be predominant in orthopedic implant-associated infections, and therefore we were particularly interested in these Gram-positive quorum-sensing molecules [19,20]. Among these, the adhesion molecule AtlE, a member of the autolysin family, mediates attachment to an implant surface in *Staphylococcus epidermidis*. Autolysins are a group of enzymes that catalyze degradation of the bacterial cell wall at specific sites and the major autolysins of *Staphylococcus aureus* (AtlA) and *Staphylocoocus epidermidis* (AtlE) have been studied in depth [20,21]. Once attached to a surface, bacteria now start to accumulate and polysaccharide intercellular adhesin (PIA) is considered to be highly important for cell-cell adhesion. PIA is a poly-β(1-6)-*N*-acetylglucosamine (PNAG), partially deacetylated, and positively charged, whose synthesis is controlled by the *icaADBC* locus and it has been shown that PIA-production positively correlated with infections associated with foreign materials [22,23,24,25].

Aside from these well-studied quorum-sensing molecules, we were interested in investigating the importance of the bacterial heat shock protein GroEL in the course of biofilm-formation. GroEL is a highly-conserved protein, which shares homologies with the human heat shock protein 60 and is essential for protein folding [26]. It has been shown in literature that bacteria cannot survive without GroEL, thus making it a strategic target for immunocompetent cells [27]. We were able to demonstrate that GroEL can be found in the extracellular polymeric substance of *Staphylococcus epidermidis* biofilms and that immune cells are able to recognize GroEL, which induces several bactericidal strategies [28,29].

The aim of this study was to selectively inhibit quorum-sensing molecules (AtlE, PIA, GroEL), that are essential for biofilm formation by specific antibodies. We used avian IgY antibodies raised against the respective QSM. Specific antibodies could offer an alternative treatment strategy to standard antibiotic therapy, because the latter is often less effective on biofilms compared to planktonic bacteria. There are several theories why an increased tolerance to antibiotics might occur, such as conventional resistance mechanisms, but also upregulated efflux pumps, mutations in antibiotic target molecules, and the possibility of a more dormant group of bacteria within the biofilm are thought to contribute [30]. For these reasons and also because an alarming rise in antibiotic resistance has been reported in general, more and more research is being dedicated towards finding alternative therapeutic options.

## 2. Results

**Figure 1:**
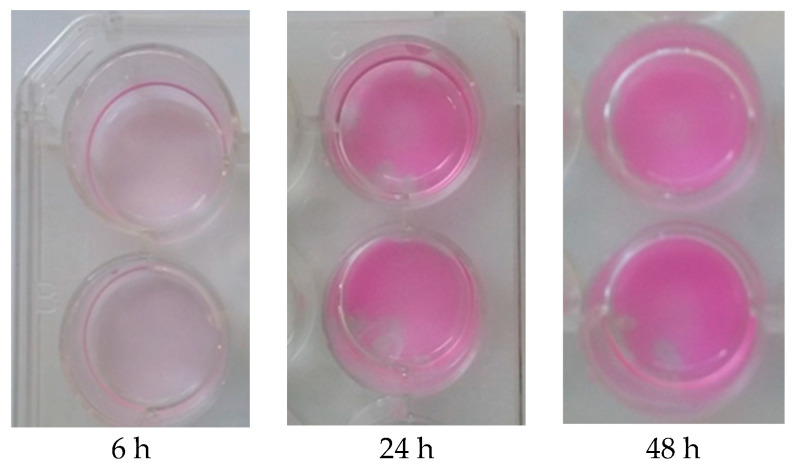
Staining of Staphylococcus epidermidis biofilms with Mira-2-ton after 6, 24 and 48 h. Bacteria were adjusted to a concentration of 3 × 10^6^/2 mL and incubated at 37 °C, shaking at 60 rpm.

**Figure 2:**
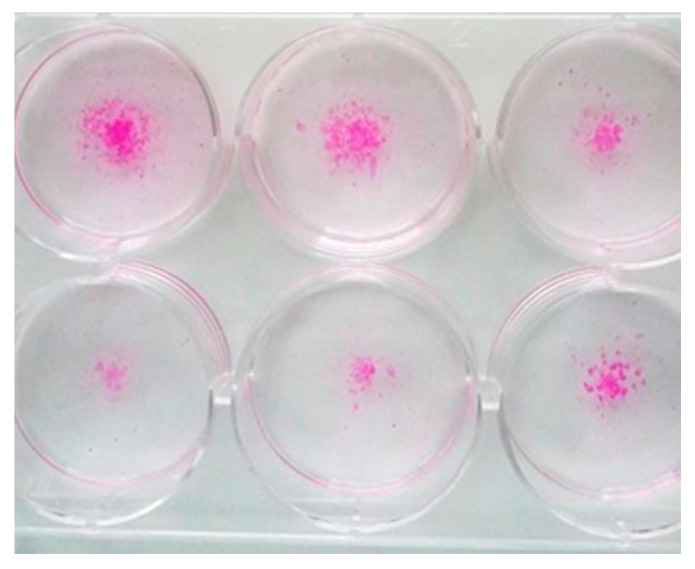
Staining of *Staphylococcus* epidermidis biofilms after 24 h incubation with IgY3 (10 µg/mL). Biofilm formation was significantly reduced when compared to results after 24 h incubation without antibodies as shown in Figure 1.

Staphylococcus epidermidis has been shown to be most commonly associated with orthopedic implant-associated infections [19]. Therefore we chose to investigate the effect of IgYs directed towards quorum-sensing molecules in St. epidermidis biofilms including AtlE (IgY1), GroEL (IgY2) and PIA (IgY3) (see Table 1).Various concentrations of bacteria and IgYs were tested at different time points and biofilms were evaluated by staining with Mira-2-Ton. Figure 1 shows staining of biofilms after 6, 24 and 48 h and Figure 2 shows reduced biofilm formation at 24 h after incubation with IgY3.

**Figure 3:**
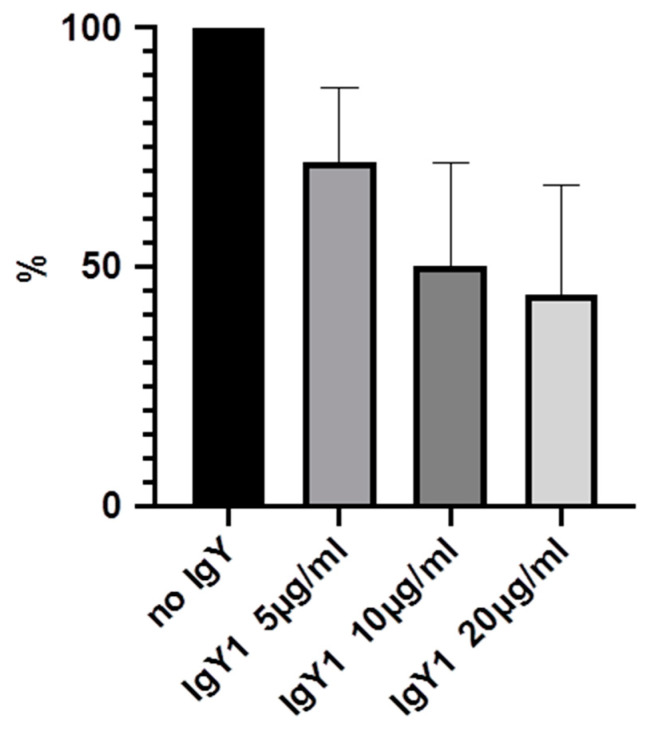
Inhibition of biofilm formation in % after 24 h incubation with 5, 10 and 20 µg/mL IgY1.

**Figure 4:**
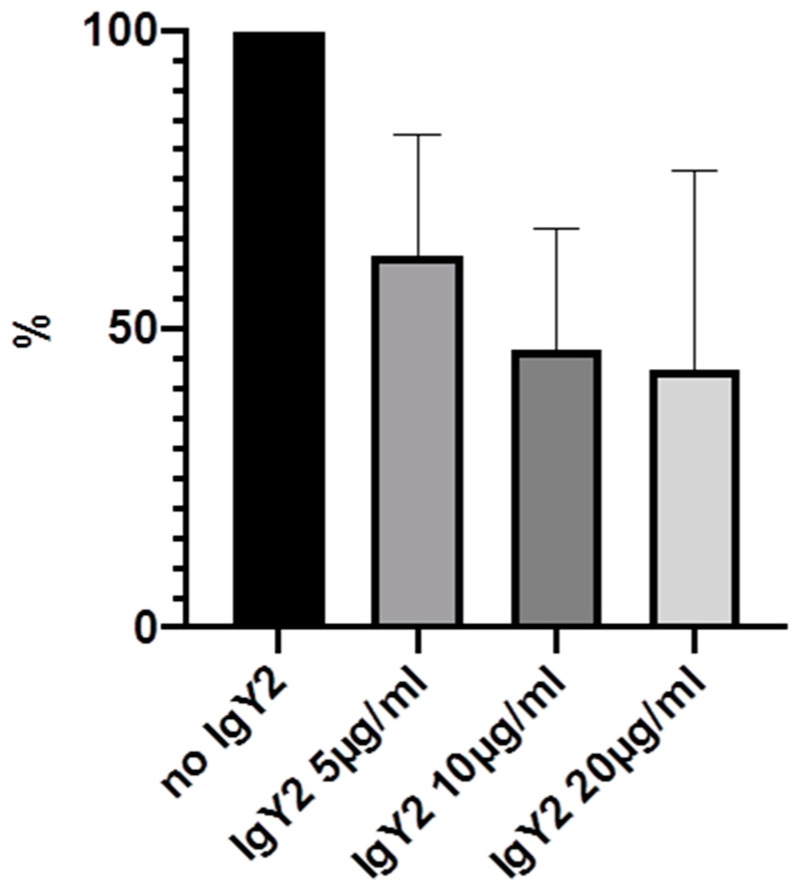
Inhibition of biofilm formation in % after 24 h incubation with 5, 10 and 20 µg/mL IgY2.

**Figure 5:**
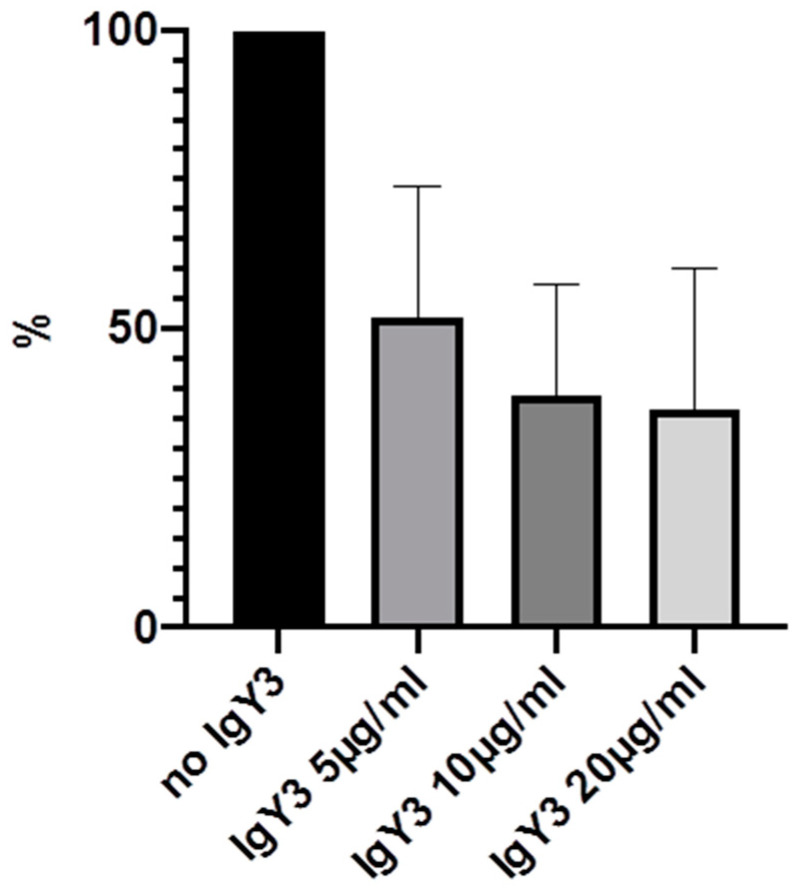
Inhibition of biofilm formation in % after 24 h incubation with 5, 10 and 20 µg/mL IgY3.

**Figure 6:**
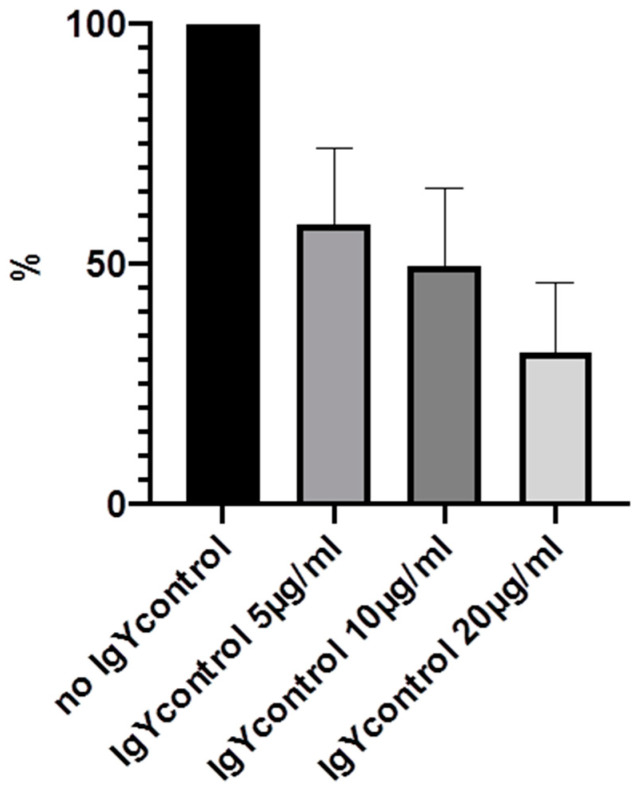
Inhibition of biofilm formation in % after 24 h incubation with 5, 10 and 20 µg/mL IgY without prior immunization.

Experiments were carried out multiple times and mean values were calculated. The highest effects could be demonstrated after 24 h, with bacterial concentration of 3 × 10^6^/2 mL and IgY-concentrations of 5, 10 and 20 µg/mL. Therefore, these results will be demonstrated in detail (Table 1 and Figure 3, Figure 4, Figure 5 and Figure 6).

We were able to demonstrate that among the three IgYs that were tested, IgY3 directed against PIA seemed to have the highest effect in reducing biofilm formation. However, there was no significant difference from the effect of IgY control, which also inhibited biofilm formation nonspecifically (*p* > 0.05, calculated with Mann-Whitney U test).

In order to evaluate biocompatibility of IgYs with human cells, primary osteoblasts were cultivated and stimulated with IgY1-3 and IgYcontrol at various concentrations. After 6 and 24 h the supernatant was removed and an ELISA analysis for IL-8, indicative of cell-activation, was performed. Furthermore, osteoblasts were also evaluated by microscopy.

**Figure 7:**
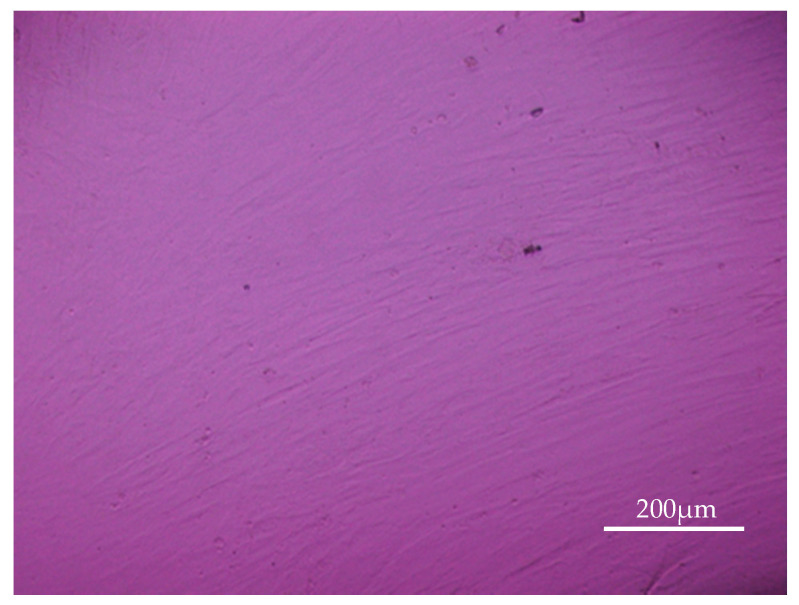
Human osteoblasts were cultivated from human bone marrow which was collected during surgery from patients who required an autologous bone graft. Cells were cultivated in osteoblast growth medium and after 10–14 days homogenous cell layers were seen. Osteoblasts were used for a maximum of two passages.

**Figure 8:**
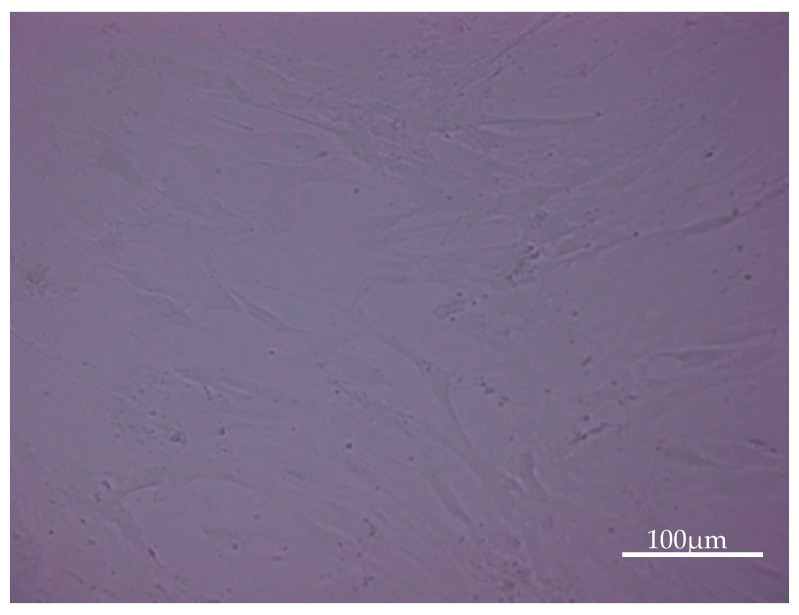
After incubation with avian IgYs for 24 h, osteoblasts were partially detached from the surface which indicates activation.

**Figure 9:**
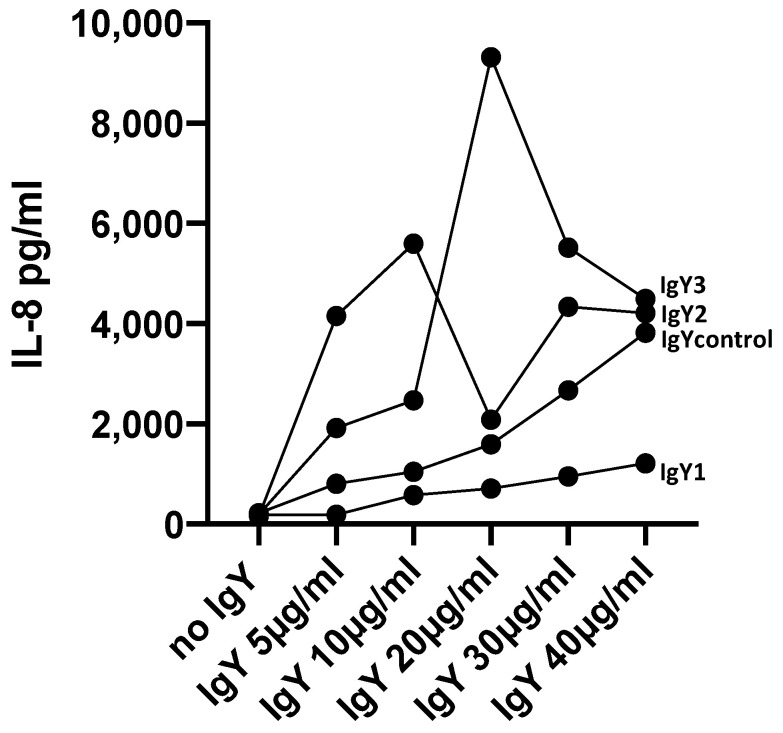
Osteoblasts were incubated with IgY1-3 and IgYcontrol at various concentrations. After 24 h supernatants were collected for IL-8 ELISA analysis. Experiments were carried out with multiple donors (*n* = 4). Individual results varied widely, but an increased release of IL-8 could be seen in all donors. This figure shows the results of one donor as an example.

After incubation with IgYs for 24 h microscopy revealed detachment of osteoblasts from the dish which indicates a negative response of osteoblasts towards IgYs (Figure 7 and Figure 8). This observation was confirmed by results from IL-8 ELISA. Osteoblasts were activated by IgYs which resulted in an enhanced release of IL-8 into the supernatant. Results of IL-8 ELISA varied widely between different donors, however an identical response was seen. IL-8 release into the supernatant increased after stimulation with IgYs. As an example, results of one donor are demonstrated in Figure 9.

## 3. Discussion

Because implant-associated infections still represent one of the major complications in orthopedic surgery, extensive research is directed towards improving diagnostics and treatment options in these cases. Once bacteria attaches to an implant surface they form a biofilm colony, which makes them increasingly difficult to detect and standard antibiotic therapy is often less effective in biofilms compared to free-swimming (planktonic) bacteria. Therefore, treatment alternatives are an area of particular interest [31,32,33,34,35]. Bacteria communicate with each other via quorum-sensing molecules in order to coordinate biofilm-formation [15]. Inhibition of these communication molecules has been proposed as a possible strategy to delay biofilm-formation [36]. This could offer immune cells additional time to fight off planktonic bacteria more easily, because defense mechanisms are impeded once a biofilm colony has been established.

The idea to impede bacterial quorum-sensing molecules has been a matter of great interest and various natural occurring inhibitors, which are produced by prokaryotes and eukaryotes alike, have been identified. This process is referred to as “quorum-quenching”. Moreover, in an effort to disrupt biofilm formation also synthetic molecules have been investigated in this context (e.g., AHL analogues) (reviewed in [37,38,39]).

The use of avian IgY has been investigated, in particular regarding infections of the digestive system [40,41,42,43]. Avian IgY resembles mammalian IgG in function, however structural differences between the two prevent activation of the complement system or other defense-relevant functions [44,45,46]. Hence, IgY has been described as a safe option to specifically target bacterial entities. Advantages of IgY also include that the immunization process of chicken is inexpensive, easy to handle and high concentrations of IgY can be extracted from the egg yolk [47].

Several reports can be found in literature, which describe using the IgY technology for several aspects in human and veterinary health, such as immunodiagnostics [48], immunotherapy [49], and bacteria [50]. Currently, clinical trials are being carried out on IgYs directed against *Pseudomonas* spp. infections in mechanically ventilated patients [51].

We were interested in investigating whether avian IgY could also be successfully directed against bacterial communication molecules in order to prevent biofilm formation on orthopedic implants. Therefore, chicken were immunized against the autolysin of *Staphylococcus epidermidis*, AtlE, which mediates attachment to a surface, against PIA, which is crucial for cell–cell adhesion in biofilms and against GroEL, the bacterial heat shock protein, which can be found in the biofilm matrix and is essential for protein folding.

Our results demonstrate that biofilm formation was reduced by all IgYs, even by IgY obtained without prior immunization. The antibody against PIA had only a minor additional inhibitory effect which was not statistically significant. Therefore, the effects of IgY on biofilm formation are apparently not specific regarding the QSM used for immunization. Naturally occurring antibodies to bacterial antigens that the animals have acquired during their life-time could account for the inhibitory effects seen.

Of note, we demonstrated that osteoblasts were affected by all of the IgYs. The cells detached from the surface and increased release of IL-8 was detected.

## 4. Materials and Methods

### 4.1. Generation of IgY

For the selection of suitable epitopes within the known protein sequence (AtlE, GroEL, PIA) of the bacteria occurring in post-operative infections, hydrophobicity and hydrophilicity scales were calculated according to Hopp-Woods and Goldman-Engleman-Steitz. These target sequences with the mathematically best antigenicity (immunogenicity for the immunization of the chickens) were selected for the antibody production. The corresponding antigen structures were synthesized in sufficient quantity using the known amino acid sequence. The quality of the peptide and a purity of at least 75% were also ensured by mass spectroscopy and HPLC analysis. Since short synthetic peptides (smaller than 10 kD) are usually not sufficiently immunogenic for the immunization of chickens, the short peptides were bound to a larger carrier protein. In this case, the respective synthesized peptide was coupled to the keyhole limpet hemocyanin (KLH, *N*-terminal modification: KLH (-NH2 of N terminal)). During the immunizations, two chickens per antigen were immunized four times within 50 days with 50 µg peptide-KLH complex.

Based on the bioinformatic analysis with the utilization of the Uniprot database it was possible to identify a 15-amino acid long target sequence within the AtlE protein. This target sequence is *glneiylishalvet* and plays a pivotal role in the carbohydrate transport and metabolism of Beta-*N*-acetylglucosaminidase. For the GroEL protein the 15-amino acid long target sequence *svaglmittecmvtd* was identified that is an active site of the ring oligomerization interface and responsible for polypeptide binding. The third target sequence *alaypyglinddrik* was generated against the active site of a poly-beta-1,6-*N*-acetyl-d-glucosamine *N*-deacetylase (for details on the chosen target sequences, see “Supplementary Materials”).

The three target sequences were synthesized by Genscript Limited (Hong Kong, China), KLH was coupled to the *N*-terminus to increase the immunogenicity of the synthesized peptides. Peptides were purified via HPLC (p1—96.8%, p2—95.7% and p3—99.0% purity) and confirmed via mass spectrometry (MW p1—1671.90, MW p2—1570.86 and MW p3—1721.96). Immunization and purification of the IgYs against the target sequences was customized and performed by Ovagen Ltd., Ballina, Ireland. Chickens were immunized separately with purified peptides in 3 intervals, starting with primary immunization (50 µg of antigen in Freud’s complete Adjuvant) and 2 boosters (two times 50 µg antigen in Freud’s complete Adjuvant (Sigma Aldrich, Darmstadt, Germany)). After 2 weeks eggs were collected and IgYs were purified from egg yolk by a two-step precipitation, then diluted in PBS pH 7.3 (Sigma Aldrich). For IgY isolation the Chicken IgY Eggspress Purification Kit was used according to the manufacturer’s instruction (Gallus Immunotech Inc., Fergus, ON, Canada). Three IgY fractions were obtained: AtlE (IgY1) (0.95 mg/mL), GroEL (IgY2) (2.9 mg/mL), and PIA (IgY3) (2.1 mg/mL) and confirmed by NanoDrop measurement at 280nm (Thermo Fisher, Bremen, Germany). Unspecific IgYs were isolated accordingly from non-immunized chickens.

### 4.2. Formation of Biofilms and Effect of IgYs

*Staphylococcus epidermidis* (RP62a, ATCC 35984) were grown overnight on a blood agar plate at 37 °C (Thermo Scientific, Wesel, Germany). The following day, bacteria were scraped of the plate, suspended in 20 mL pre-warmed Trypticase Soy Broth (TSB) and a pre-culture was performed for 90 min at 37 °C. Bacteria were then adjusted to the following concentration in 2 mL TSB: 7.5 × 10^5^, 2 × 10^6^, 3 × 10^6^, 2 × 10^7^. Experiments were carried out in 6-well culture dishes (NuncTM, Wiesbaden, Germany). The following concentrations of IgY1-3 and IgYcontrol were added: 5, 10, 20, 30, 40 µg/mL. Culture dishes were incubated at 37 °C, shaking at 60 rpm. After 6, 24 and 48 h the supernatant was removed, biofilms were carefully washed with 0.9% NaCI and then stained with Mira-2-Ton (Miradent, Hager&Werken GmbH, Duisburg, Germany). After 3 min biofilms were again washed with 0.9% NaCI. Then, 2 mL of propanol 50% was added and the color reaction was measured by optical density (OD540nm). Experiments were carried out multiple times.

### 4.3. Culture of Osteoblasts

Primary osteoblasts were cultivated from human bone marrow which was harvested either from the femoral bone using the RIA (reamer-irrigator-aspirator)-technique or from the iliac crest of patients undergoing surgery due to fracture malunion or nonunion, and who required an autologous bone graft. Informed consent was obtained from the patients, and the study was approved by the local ethic committee of Heidelberg University (S-355/2010). Samples were grinded using sterile scalpels and cultivated in osteoblast growth medium (PromoCell, Heidelberg, Germany) containing 0.5% penicillin/streptomycin (Gibco Life Technologies, Eggenstein, Germany). Outgrowth of cells occurred usually between 4 to 8 days. Cells were subcultivated following digestion with trypsin (0.05% Trypsin-EDTA, Life Technologies) for 5 min at 37 °C and resuspended in osteoblast growth medium. After 10 to 14 days, homogenous cell layers were seen. Osteoblasts were used for a maximum of two passages and experiments were carried out in 24-well dishes (NuncTM, Wiesbaden, Germany) at a concentration of 2 ×  10^4^ cells/mL in osteoblast growth medium.

### 4.4. Bicompatibility of IgY

Osteoblasts were stimulated with IgY1-3 and IgYcontrol with the following concentrations: 5, 10, 20, 30, 40 µg/mL. After 6 and 24 h supernatants were collected for ELISA analysis and osteoblasts were evaluated by microscopy.

### 4.5. ELISA

IL-8 in cell culture supernatants were determined using commercially available ELISA kits according to the protocol provided by the manufacturer. The human ELISA kits were purchased from R&D Systems (Minneapolis, MN, USA).

### 4.6. Statistical Analysis

Differences between groups were calculated using Mann-Whitney test using GraphPad, Prism8 software. Significance level was determined as *p* <  0.05.

## 5. Conclusions

In summary, we were able to show that biofilm formation can be reduced by avian IgY. The target of the IgY has not yet been identified, so the mechanism of inhibition is not yet clear. While the idea of inhibiting biofilm formation by antibodies is intriguing, IgY is possibly not the best choice because of its adverse effects on osteoblasts.

## Figures and Tables

**Table 1:** Inhibition of biofilm formation in % by IgY1–3 and IgYcontrol. Mean values of 5 experiments and standard deviation are shown.

	No IgY	5 µg/mL (SD)	10 µg/mL (SD)	20 µg/mL (SD)
IgY1 (AtlE)	100%	72% (15.4)	50.2% (21.7)	44.4% (22.7)
IgY2 (GroEL)	100%	62.3% (20.3)	46.8% (19.9)	43.3% (33.3)
IgY3 (PIA)	100%	52% (21.8)	38.8% (18.5)	36.6% (23.4)
IgYcontrol	100%	58.4% (15.8)	49.6% (16.2)	31.8% (14.4)

## Supplementary Materials

The following are available online.
