# Supplementary material for: IgY Targeting Bacterial Quorum-Sensing Molecules in Implant-Associated Infections
Source: Molecules. 2020 Sep 3;25(17):4027. doi: 10.3390/molecules25174027 (PMC7504788; doi:10.3390/molecules25174027)
Supplement: Supplementary file 1 [file molecules-25-04027-s001.pdf]

Supplementary Materials for

IgY Targeting Bacterial Quorum-Sensing Molecules in Implant-Associated Infections

chosen target sequences:

**AtlE**	LOCUS ATL_STAEP 1335 aa linear BCT 28-MAR-2018<br>DEFINITION RecName: Full=Bifunctional autolysin; AltName: Full=AtlE; Includes: RecName: Full=*N*-acetylmuramoyl-L-alanine amidase; Includes:<br>RecName: Full=Mannosyl-glycoprotein<br>endo-beta-N-acetylglucosaminidase; Flags: Precursor.<br>ACCESSION O33635<br>VERSION O33635.1<br>DBSOURCE UniProtKB: locus ATL_STAEP, accession O33635;<br>Secreted;<br>Signal.<br>SOURCE Staphylococcus epidermidis<br>1081 gkyvwikstd lvkekikyay tgmtlnnain iqsrlkykpq vqneplkwsn anysqiknam<br>1141 dtkrlandss lkyqflrldq pqylsaqaln kllkgkgvle nqgaafsqaa rkyglneiyl<br>1201 **ishalvet**gn gtsqlakggd vskgkfttkt ghkyhnvfgi gafdnnalvd gikyaknagw<br>1261 tsvskaiigg akfignsyvk agqntlykmr wnpanpgthq yatdinwanv naqvlkqfyd
**GroEL**	LOCUS CH60_ECOLI 548 aa linear BCT 28-MAR-2018<br>DEFINITION RecName: Full=60 kDa chaperonin; AltName: Full=GroEL protein; AltName: Full=Protein Cpn60.<br>ACCESSION P0A6F5<br>VERSION P0A6F5.2<br>DBSOURCE UniProtKB: locus CH60_ECOLI, accession P0A6F5;<br>421 rvaskladlr gqnedqnvgi kvalrameap lrqivlncge epsvvantvk ggdgnygyna<br>481 ateeygnmid mgildptkvt rsalqyaasv aglmittecm vtdlpkndaa dlgaaggmgg<br>541 mggmggmm
**PIA**	LOCUS AAQ88122 289 aa linear BCT 29-APR-2005<br>DEFINITION IcaB [Staphylococcus epidermidis].<br>ACCESSION AAQ88122<br>VERSION AAQ88122.1<br>DBSOURCE accession AY382582.1<br>KEYWORDS .<br>SOURCE *Staphylococcus* epidermidis<br>ORGANISM *Staphylococcus* epidermidis<br>Bacteria; *Firmicutes; Bacilli; Bacillales; Staphylococcaceae; Staphylococcus.*<br>REFERENCE 1 (residues 1 to 289)<br>AUTHORS Li,H., Xu,L., Wang,J., Wen,Y., Vuong,C., Otto,M. and Gao,Q.<br>TITLE Conversion of *Staphylococcus* epidermidis strains from commensal to invasive by expression of the ica locus encoding production of<br>biofilm exopolysaccharide<br>121 dmdqtiydna spvlkkyhip atgflitnhi gstnfhnlnl lskkqldemy etglwdfesh<br>181 thdlhalkkg nkskfldssq svaskdikks ehylnknypk neralaypyg linddrikam<br>241 kkngiqygft lqekavtpda dnyriprilv sndafetlik ewdgfdeek
